# A Hard Lump to Chew: Delayed Diagnosis of Foreign Body Aspiration in an Elderly Man Leading to Severe Respiratory Failure

**DOI:** 10.7759/cureus.91304

**Published:** 2025-08-30

**Authors:** Vera Vieira, Jorge Mendes, João Machado, Nuno Ferreira, Salvato Feijó

**Affiliations:** 1 Intensive Care Unit, Unidade Local de Saúde da Região de Leiria, Leiria, PRT; 2 Pulmonology Department, Unidade Local de Saúde da Região de Leiria, Leiria, PRT; 3 Intensive Care Unit, Instituto Português de Oncologia de Coimbra Francisco Gentil, Coimbra, PRT; 4 Critical Care Medicine, Centro Hospitalar de Leiria, Leiria, PRT

**Keywords:** elderly, foreign body aspiration, intensive care, respiratory failure, rigid bronchoscopy

## Abstract

Foreign body aspiration (FBA) in adults is uncommon and often associated with risk factors such as advanced age and chronic alcohol abuse. It can constitute a serious emergency with acute symptoms secondary to airway obstruction, but there are cases in which it goes unnoticed, causing late complications with insidious symptoms and recurrent respiratory infections. We present the case of a 77-year-old male patient with a history of hypertension, type 2 diabetes mellitus, dyslipidemia, hyperuricemia, probable chronic obstructive pulmonary disease, chronic alcohol abuse, and gonarthrosis, who was admitted with acute respiratory failure. Initial diagnosis suggested hypertensive pulmonary edema; however, chest computed tomography revealed a foreign body in the right lower lobe bronchus. Flexible bronchoscopy confirmed the presence of the foreign body but failed to remove it. Subsequent rigid bronchoscopy successfully extracted an olive pit. The patient required mechanical ventilation and intensive care support but eventually recovered. This case underscores the importance of considering FBA in adults presenting with unexplained respiratory symptoms, especially those with predisposing factors.

## Introduction

Foreign body aspiration (FBA) can be a serious and potentially fatal event [1]. Although more frequent in children, it also affects adults, particularly those with risk factors such as advanced age, neurological compromise (altered consciousness, dysphagia), alcohol abuse, and facial trauma [2-4]. A higher incidence in males has also been reported [2]. Clinical presentations range from acute airway obstruction requiring immediate intervention to chronic, indolent symptoms such as cough, hemoptysis, mild dyspnea, wheezing, fatigue, and chest pain [1,4-6]. Some patients may remain asymptomatic for extended periods. Delayed diagnosis increases the risk of complications, including granulation tissue formation, recurrent pneumonia, lung abscess, atelectasis, bronchial stenosis, pneumothorax, and pneumomediastinum [1,6,7].

A high index of suspicion is essential, particularly in cases of recurrent pneumonia or persistent respiratory symptoms [2]. Chest X-ray has limited sensitivity (28-60%) and specificity (68%) [6], and is often normal in cases involving organic materials [1,3,4,6]. Computed tomography (CT) remains the gold standard for diagnosis [7], with common findings including unilateral hyperlucency, bronchiectasis, atelectasis, lobar consolidation, and pleural effusion [4,7]. The right bronchial tree, particularly the right intermediate bronchus, is the most frequent site of impaction due to anatomical factors [2,3,5-7].

Both flexible and rigid bronchoscopy are viable options for foreign body removal. Flexible bronchoscopy is widely available, requires no sedation in cooperative patients, but poses challenges in maintaining airway patency. It achieves successful removal in over 90% of adult cases [1,7]. Rigid bronchoscopy allows better airway control and simultaneous instrument use but requires sedation, technical expertise, and is less accessible [4,7]. In failed flexible attempts or delayed diagnoses, rigid bronchoscopy is preferred. It is the first-line approach in cases of asphyxiation. For stable patients, flexible bronchoscopy by experienced teams remains a reasonable first step, with conversion to rigid technique as needed [4,7]. Surgical intervention is rarely required [3,7].

## Case presentation

A 77-year-old man with a medical history of hypertension, type 2 diabetes mellitus (non-insulin-dependent), dyslipidemia, hyperuricemia, unstudied structural lung disease, smoking habits, chronic alcohol abuse, and gonarthrosis presented to the emergency department with resting dyspnea. Upon arrival, he exhibited signs of respiratory exhaustion, tachycardia, and hypertension. Initial assessment suggested hypertensive pulmonary edema, and non-invasive ventilation (NIV) was initiated without significant improvement. The patient was transferred to a tertiary care hospital for further management.

**Figure 1:**
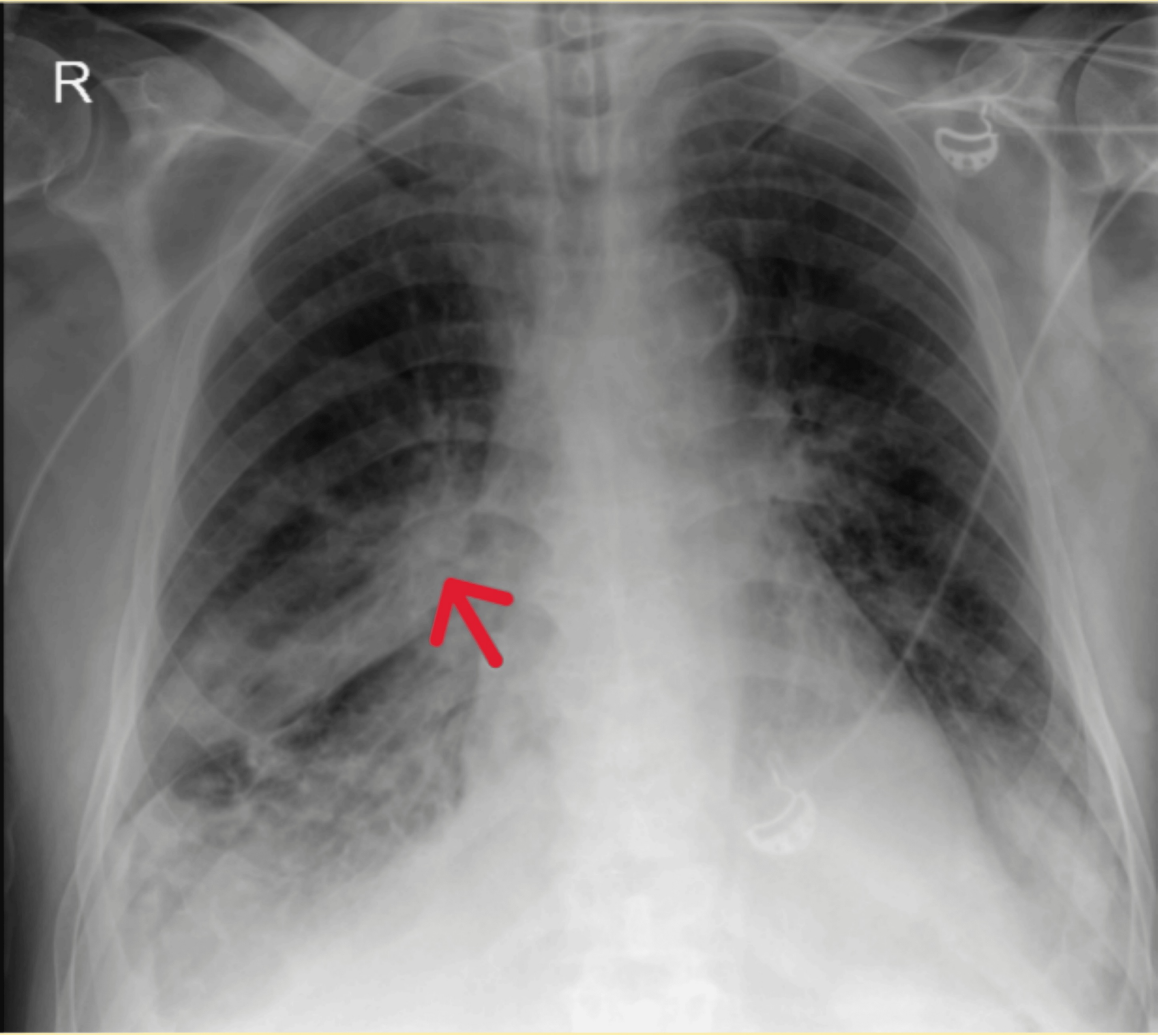
Chest X-ray with opacity of the right middle and lower lobe (red arrow)

On admission, he was tachypneic with shallow breathing, oxygen saturation of 88-89% on BiPAP (IPAP 20, EPAP 10, FiO₂ 45%), and a respiratory rate of 34-38 breaths per minute. Chest auscultation revealed diffuse crackles. Chest radiograph indicated decreased transparency in the right lung base, suggestive of consolidation (figure 1). Empirical antibiotic therapy with amoxicillin-clavulanic acid was initiated. Arterial blood gas analysis showed pH 7.29, pO₂ 62 mmHg, pCO₂ 45 mmHg, HCO₃⁻ 21.6 mmol/L, and lactate 2.0 mmol/L.

Due to worsening respiratory effort and inadequate gas exchange, endotracheal intubation was performed following rapid sequence induction. Mechanical ventilation was initiated, and sedation was maintained with propofol and fentanyl. The patient developed hypotension unresponsive to fluid challenge, and norepinephrine infusion was started for vasopressor support. An echocardiogram was performed, which did not reveal any significant impairment of function.

**Figure 2:**
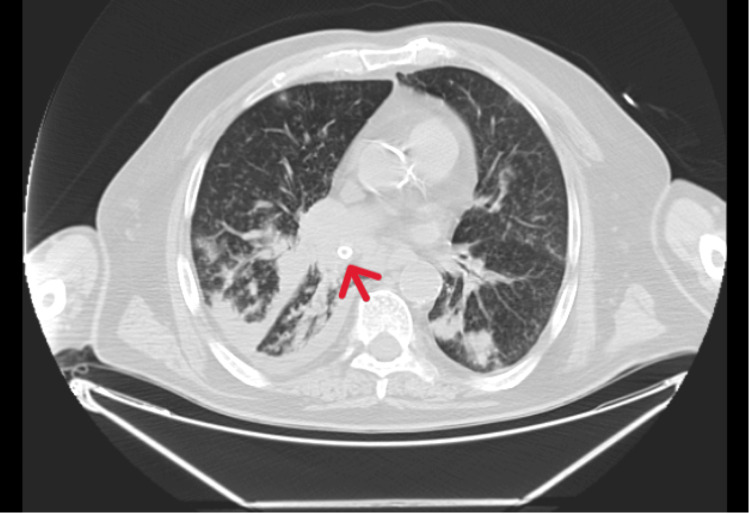
Chest CT scan showing mucous filling of the bronchial tree in the basal segments of the right lower lobe, indicating the presence of an oval structure with coarse peripheral calcification (red arrow), apparently in an intraluminal location in the respective lobar bronchus with subtotal pulmonary atelectasis of this lung lobe

**Figure 3:**
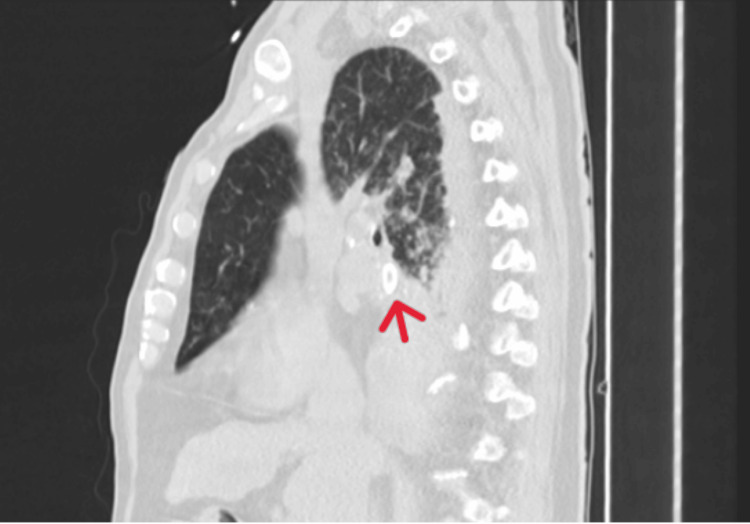
Coronal plan of the chest CT scan showing an oval structure with coarse peripheral calcification, with approximately 19 mm of longest axis (red arrow)

Before admission to the ICU, chest computed tomography was performed and revealed a possible endobronchial foreign body in the right lower lobe bronchus-an oval-shaped, calcified structure (~19 mm), causing subtotal lobar atelectasis (Figures 2,3). 

The patient was admitted to the ICU with septic shock secondary to community-acquired pneumonia, suspected to be related to FBA, with respiratory, cardiovascular, and renal dysfunction.

**Figure 4:**
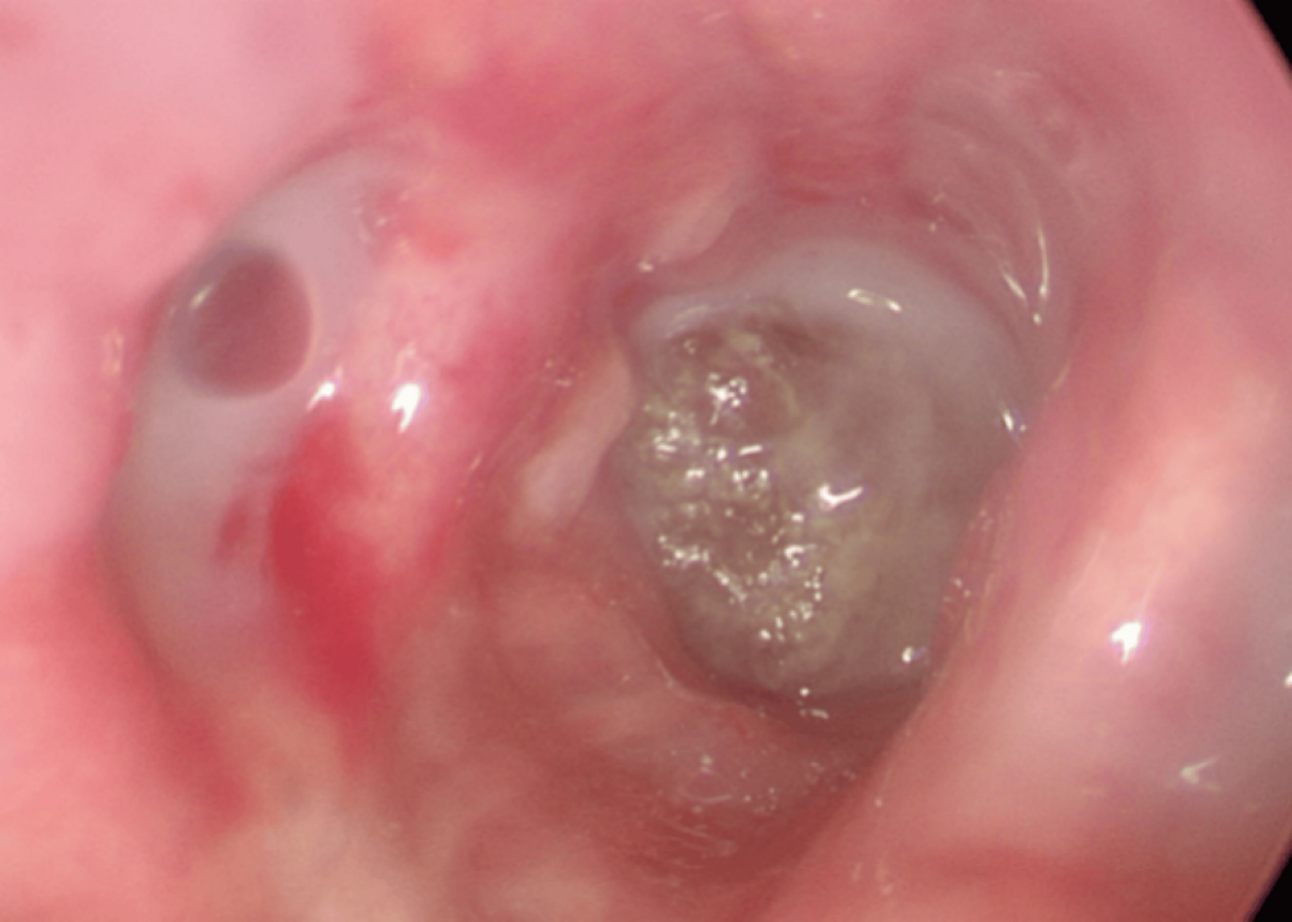
Rigid bronchoscopy revealing a foreign body (olive pit) surrounded by abundant purulent secretions embedded in the trunk of the right basal pyramid, which was removed with forceps. The rest of the bronchial tree showed abundant purulent secretions.

**Figure 5:**
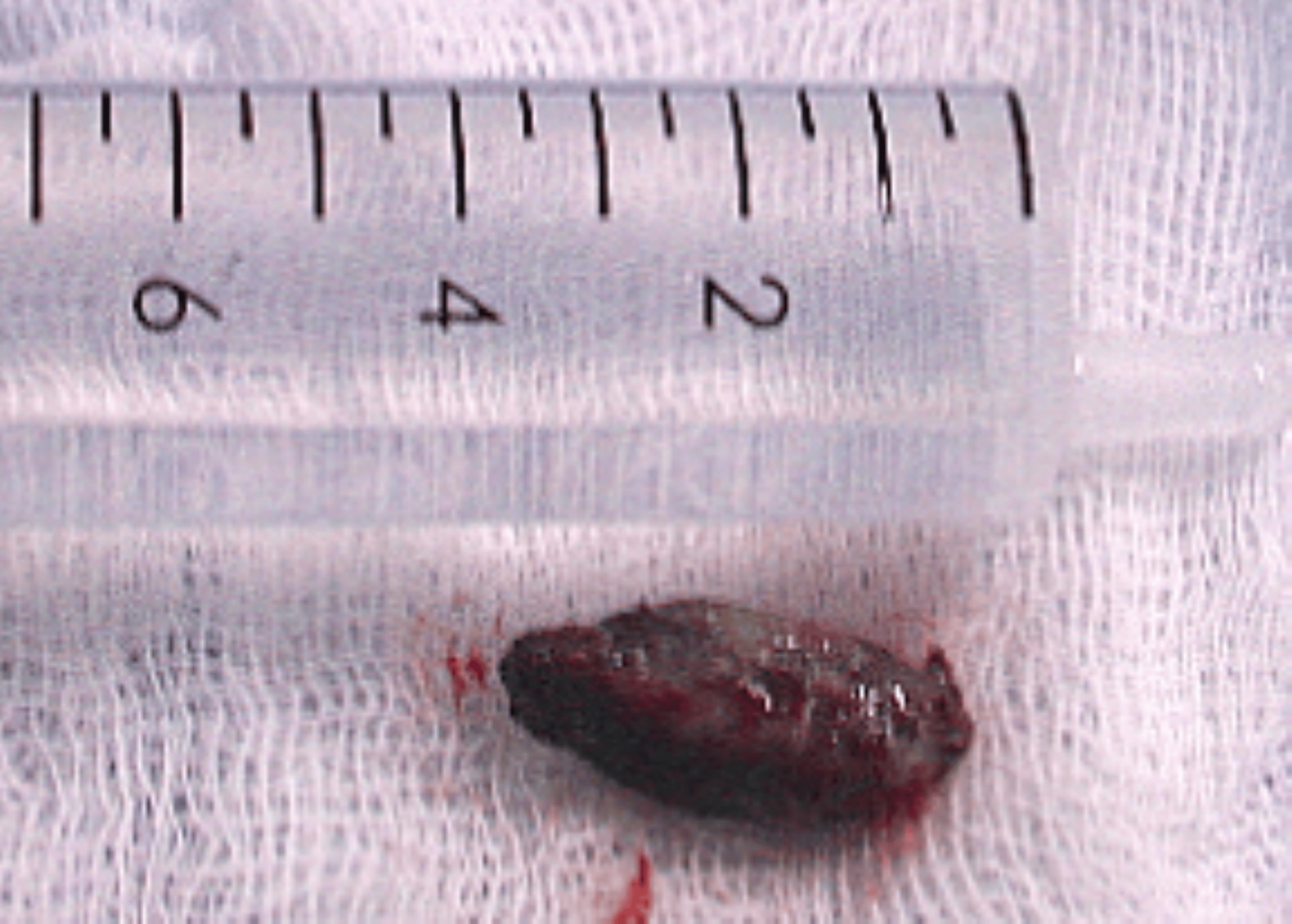
Olive pit removed

Flexible bronchoscopy confirmed the presence of an endobronchial foreign body, but removal was unsuccessful. The following day, rigid bronchoscopy was performed, successfully extracting an olive pit embedded in purulent secretions from the right lower bronchial pyramid (Figures 4,5). Copious purulent secretions were noted throughout the bronchial tree.

Post-removal, the patient required frequent aspiration of mucopurulent secretions. Repeat flexible bronchoscopy showed moderate secretions without mucosal abnormalities. The patient was extubated on Day 6 without complications and briefly required high-flow nasal cannula support due to worsening gas exchange. At discharge, he was eupneic on nasal oxygen. He completed a seven-day course of amoxicillin-clavulanic acid and five days of clarithromycin. Enterobacter cloacae resistant to ampicillin and amoxicillin-clavulanic acid was isolated in bronchoalveolar lavage, but given clinical and imaging improvement post-removal, no antibiotic escalation was needed. He was transferred to the Pulmonology department for further management.

## Discussion

FBA in adults is rare. It represents only 15-25% of all FBA cases [8], but potentially life-threatening and often associated with risk factors such as advanced age and chronic alcohol abuse [2,3,7]. As with our patient, clinical presentation in adults is often subtle and nonspecific-including persistent cough, chronic respiratory symptoms, or even findings mimicking malignancy-which may delay recognition and contribute to complications [1,2,4-7].

Many aspirated objects are radiolucent, especially organic materials such as food particles, wood, or thin bones, and therefore may not be visible on chest radiographs [1,3,4,7]. In such cases, CT is more sensitive and is increasingly regarded as the imaging modality of choice for localization [3,7]. CT substantially increases detection of radiolucent substances and associated complications such as bronchiectasis, atelectasis, infection and granulation tissue. In cases of high clinical suspicion with normal X-rays, CT should be performed [3,4,7].

Regarding interventional strategies, the role of bronchoscopy has evolved. Flexible bronchoscopy has become the initial procedure of choice in many centers, particularly in stable patients [4,7]. Reported success rates range between 60% and 100%, with large retrospective series showing an overall efficacy close to 90% [1]. It offers the advantage of being less invasive, often performed under moderate sedation [4].

Systematic reviews, although mostly of pediatric populations, suggest comparable success rates between flexible and rigid techniques, but note that flexible bronchoscopy is associated with less desaturation and shorter hospitalization, at the expense of a higher conversion rate to rigid bronchoscopy [4,7].

In our case, flexible bronchoscopy was unsuccessful in removing the foreign body, which is consistent with many case reports, specialy in delayed diagnosis which, frequently, lead to granulation tissue formation and foreign body impactation. This increases the probability of flexible bronchoscopy flailure and highlights the importance of rigid bronchoscopy in such scenarios [1,4,5,7]. When flexible bronchoscopy fails, rigid bronchoscopy remains highly effective. Its advantages include superior airway control, larger suction capacity, and the use of sturdier extraction tools [1,7].

The use of rigid bronchoscopy as the definitive procedure in our patient aligns with the litterature in which rigid bronchoscopy appears as the procedure of choice for embedded, large or hard foreign bodies [1,3-7]. Thoracotomy is rare but still reported in stubborn or complicated cases [3].

Taken together, these findings support a stepwise approach: flexible bronchoscopy as a first-line intervention in stable patients, with rigid bronchoscopy reserved for failed attempts or high-risk scenarios. Prompt diagnosis and timely intervention remain critical to reducing the morbidity and mortality associated with adult FBA.

## Conclusions

This case highlights the importance of considering FBA as a differential diagnosis in patients presenting with recurrent or non-resolving pneumonia, especially when clinical and radiological findings are atypical or unresponsive to standard treatment. Prompt recognition and bronchoscopic removal of the foreign body are crucial to prevent long-term complications and ensure complete resolution of symptoms. Clinicians should maintain a high index of suspicion, particularly in patients with risk factors or unexplained respiratory symptoms.
